# Mycotic aortic aneurysm in a debilitated patient with compromised immunity; beware of Candida!

**DOI:** 10.1590/1677-5449.210122

**Published:** 2021-12-01

**Authors:** Thilina Gunawardena, Manujaya Godakandage, Sachith Abeywickrama, Rezni Cassim, Mandika Wijeyaratne

**Affiliations:** 1 The Royal Liverpool University Hospital, Liverpool, the United Kingdom.; 2 National Hospital of Sri Lanka, Colombo, Sri Lanka.; 3 Worcestershire Acute Care Hospitals, the United Kingdom.

**Keywords:** malignant otitis externa, candida, mycotic aneurysm, in situ repair, otite externa maligna, cândida, aneurisma micótico, reparo *in situ*

## Abstract

Candida is a rare cause of infected aortic aneurysms. We report the case of a diabetic patient with end stage kidney disease who underwent repair of a leaking abdominal aortic aneurysm. He was on long-term antibiotic treatment for malignant otitis externa. *Candida albicans* was isolated from the culture of the excised aneurysm wall. An infected aortic aneurysm due to Candida has not been previously reported in a patient with malignant otitis externa. This case report aims to highlight that Candida should be suspected as a cause of infected aortic aneurysms in patients with debilitation and chronic immunosuppression. Management of such cases can be extremely challenging, especially in resource-poor settings, and we will be touching upon the advantages and disadvantages of various treatment options.

## Resumo

A cândida é uma causa rara de aneurismas da aorta infecciosos. Relatamos o caso de um paciente diabético com doença renal terminal, que foi submetido a reparo de aneurisma da aorta abdominal com vazamento. Ele estava em tratamento de longo prazo com antibióticos para otite externa maligna. A *Candida albicans* foi isolada da cultura da parede do aneurisma que sofreu a excisão. Não há relatos prévios de aneurisma da aorta infeccioso causado por cândida em pacientes com otite externa maligna. Este relato de caso visa reforçar que a cândida deve ser uma das suspeitas de causa de aneurisma da aorta infeccioso em pacientes debilitados e com imunossupressão crônica. O manejo desses casos pode ser extremamente desafiador, principalmente em contextos em que os recursos são escassos, e mencionaremos as vantagens e desvantagens das diversas opções de tratamento.

## INTRODUCTION

Mycotic aneurysms are caused by infective degeneration of the vessel wall. Although the name implies fungi as the source of such infections, this is a misnomer, and fungi are rarely isolated as the causative organism. Invasive candida infection is associated with risk factors such as immune deficiency, critical illness, and prolonged antibiotic exposure.^1^ Malignant otitis externa (MOE) is an invasive infection involving the external auditory canal (EAC) and the lateral skull base.^2^ It is a condition that is seen in diabetics, the elderly, and the debilitated.^3^ In this case report, we present a patient with MOE who was diagnosed with a leaking abdominal aortic aneurysm (AAA). He was operated on, and in situ repair of the aneurysm was done using a bifurcated polyester graft. The culture of the aneurysm wall grew *Candida albicans*. Mycotic AAA due to *Candida* spp. is rare and has never been reported in a patient who was on long term antibiotics for MOE.

The study was conducted in accordance with the relevant standards of the institutional ethics committee and the Helsinki declaration. Informed written consent was obtained from the relatives before data collection and publication of the case report.

## CASE DESCRIPTION

**Figure 1:**
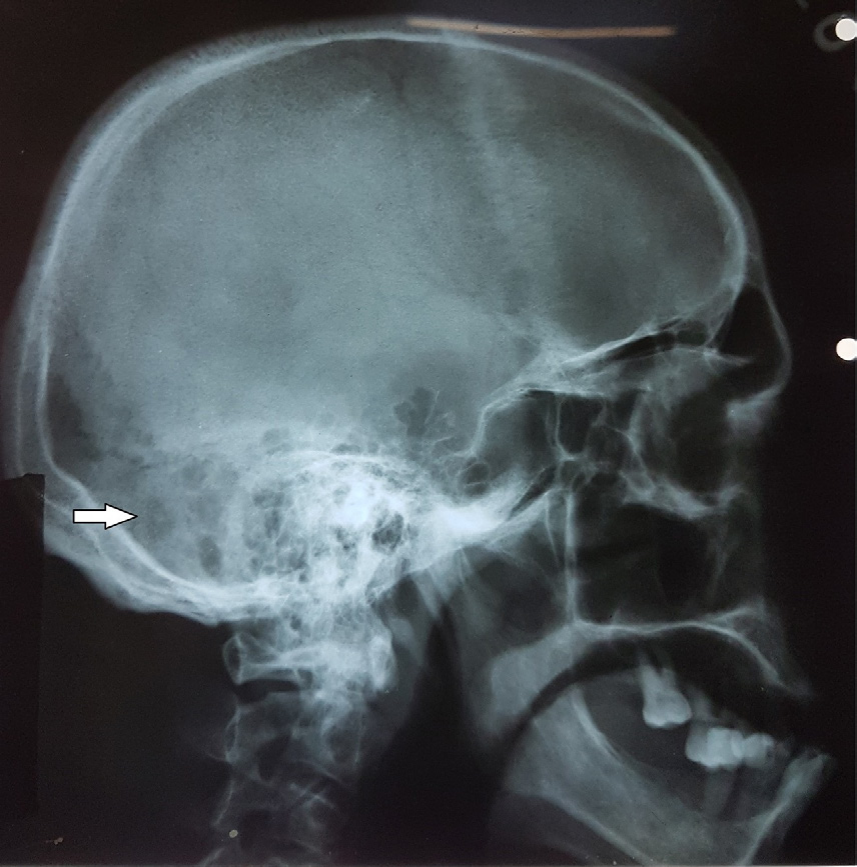
Lateral skull X-ray showing bony erosions (Arrow) suspicious for osteomyelitis.

A 67-year-old diabetic, pre-dialysis patient with end-stage kidney disease (ESKD) secondary to diabetes nephropathy on top of a single functioning kidney was investigated for a severely painful, discharging right ear. The initial pus swab was positive for pseudomonas and he was treated with intravenous ceftazidime. Despite prolonged antibiotic therapy he continued to suffer from persistent symptoms. An excision biopsy of a polyp at the bony-cartilaginous junction of the external ear canal revealed granulation tissue with focal suppuration. Following the biopsy results and a skull X-ray which was suspicious for osteomyelitis (Figure 1), a clinical diagnosis of MOE was made. Intravenous antibiotics were continued until cultures became negative and inflammatory parameters stabilized. Then he was discharged on oral ciprofloxacin. During the following year, he had 2 hospital admissions with otorrhea and otalgia. Repeated cultures of the ear discharge were negative.

**Figure 2:**
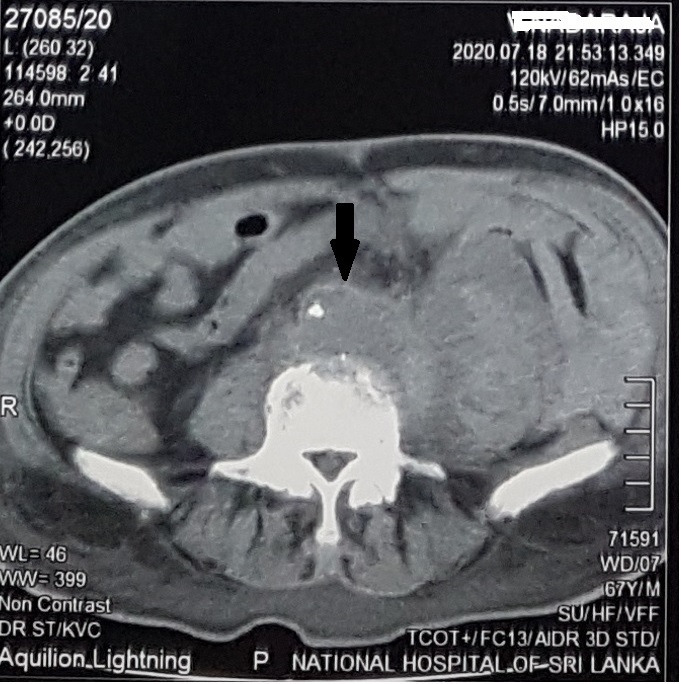
Non contrast CT findings (Arrow indicates the aneurysmal aortic bifurcation).

While in hospital for an exacerbation of ear pain, the patient developed sudden onset, severe, left-sided lower abdominal pain. On examination, his pulse rate was 112 per minute and blood pressure was 170/100 mmHg. There was a pulsatile, tender mass in the left lower abdomen clinically suggestive of an aneurysm. An urgent ultrasound scan (USS) showed an infrarenal AAA with an anterior-posterior diameter of 4.2cm. Adjacent to the aneurysm, there was a large retroperitoneal hematoma indicative of leaking. A non-contrast CT of the abdomen confirmed the USS findings. (Figure 2) Investigations available at the time revealed a WBC count of 13.26 x 10^3^ µL, Hb of 7.7g/dL, creatinine of 4.77mg/dL, C reactive protein level of 156mg/dL, and an ESR of 130mm in the first hour.

**Figure 3:**
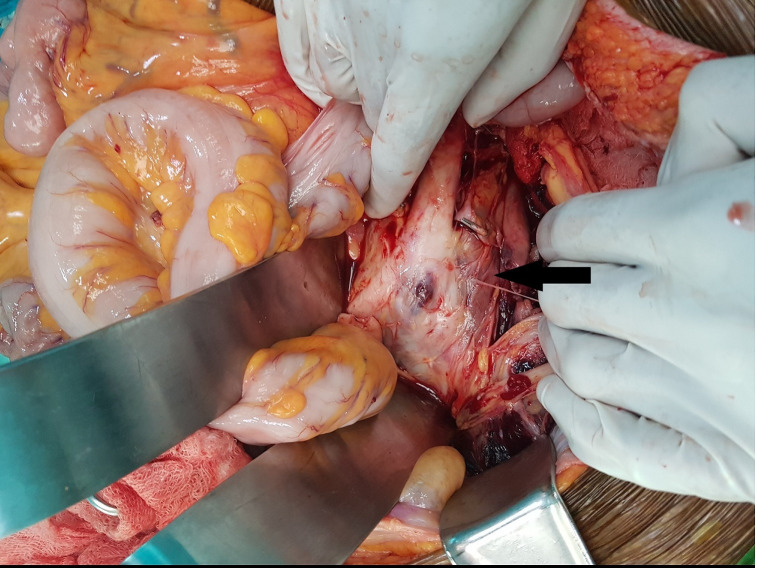
Intra-operative findings (Arrow indicates the aneurysmal aortic bifurcation).

The patient was rushed to the theater and a midline laparotomy was done, which revealed a retroperitoneal hematoma with an aneurysm at the aortic bifurcation. The appearance of the aneurysm was suspicious for infective arteritis. (Figure 3). Proximal control of the aorta was obtained below the renal artery level. The right common iliac artery (CIA) was clamped just above its bifurcation. As the left CIA had been destroyed by the inflammatory process, the left internal iliac artery was sacrificed and left external iliac artery (EIA) control was taken. The degenerated arterial wall and the adjacent left psoas muscle were debrided. Pieces from the vessel wall were taken for culture and histology. Arterial continuity was restored by in situ repair using a 14 x 7mm bifurcated polyester vascular graft. The right graft limb was anastomosed to the right CIA and the left graft limb to the left EIA. Total intraoperative blood loss was 3.5L. The patient was sent to the intensive care unit (ICU) for post-operative care.

During the ICU stay, the patient became anuric and continuous renal replacement therapy was initiated. The culture of the aneurysm wall yielded a pure growth of *Candida albicans* and intravenous fluconazole was started. Blood cultures were negative. The histology of the aortic wall was reported as degenerated vessel wall with thrombosis and suppuration. The postoperative recovery was complicated with ventilator associated pneumonia (VAP) with a multidrug-resistant *Acinetobacter* species. It was difficult to mobilize the patient for a 2D echo to rule out infective endocarditis as he was ventilator dependent. He passed away in the ICU on post-operative day 13.

## DISCUSSION

Malignant otitis externa has been defined as an invasive infection involving the EAC and the lateral skull base.^2^ As per Cohen et al.,^3^ a diagnosis of MOE can be established when there is pain, swelling, discharge, granulation tissue, microabscess, and a positive Technetium 99 bone scan. When a bone scan is unavailable, poor response to a trial of antibiotics for 1-3 weeks can be taken as an alternative criterion. Those affected with MOE are usually elderly and immunocompromised. The condition can be clinically suspected when there is ear pain disproportionate to examination findings. *Pseudomonas aeruginosa* is the most common organism isolated from patients with MOE. The mainstay of management of this condition is prolonged antibiotic therapy.^2^

Infected aneurysms are termed ‘mycotic aneurysms’. The name is derived from the mushroom-like appearance of the infected vessel wall.^4^ The majority have bacterial etiology. In only 1-2% of cases, fungi are identified as the responsible organism.^4^^,^ 5 Mycotic aortic aneurysms due to *Candida* spp. are rare, and the literature is limited to a handful of case reports.

Invasive candida infections are seen in immunocompromised and/or critically ill patients.^1^ In some patients with candida aortitis, a primary focus for the infection can be identified. Endocarditis, central line infection, and colonoscopic polypectomy are examples reported in the literature.^6^^-^ 8 In MOE, a culture of the ear discharge may grow fungal species including *Candida*.^2^ However, our patient never had an ear swab positive for candida. His blood culture taken after the aneurysm repair was negative. We could not completely rule out the possibility of fungal endocarditis as he expired before an echocardiogram and the relatives declined the request for a post-mortem examination. Our patient was a high-risk candidate for invasive candidiasis due to the presence of diabetes and ESKD, which are known risk factors for a sub-optimal immune response, and the history of prolonged broad spectrum antibiotic therapy.^1^^,^ 9 ^,^ 10

The standard treatment for a mycotic aortic aneurysm is extra-anatomic reconstruction and appropriate antimicrobial therapy.^11^ Tunneling the vascular graft away from the infected field does not completely exclude the risk of graft infection as seeding of the organism can occur through the bloodstream. Compared to extra-anatomic bypass, in situ reconstruction is associated with better long-term patency but a higher incidence of graft infection. In their case series, Dubois et al.^11^ reported an 8% reinfection rate after in situ reconstructions of infected aortic aneurysms. When in situ repair is contemplated for a mycotic aneurysm, radical debridement of all infected tissue is mandatory.^12^^,^ 13 Prosthetic grafts can be avoided if alternatives such as the patient’s femoral veins or cryopreserved aortic allografts can be used. Harvesting and preparation of femoral veins increase the surgical time and tissue trauma, which can prove detrimental in a physiologically unstable patient with a leaking AAA. Cryopreserved allografts avoid these issues, but they may not be available on an emergency basis and can be prone to aneurysmal degeneration on long-term follow-up.^14^ If a prosthetic has to be used, antibiotic-impregnated grafts are preferred.^12^^,^ 15

Endovascular stenting is a useful technique to manage mycotic aneurysms, and it has been used with success to treat a mycotic AAA caused by *Candida* as well.^16^^,^ 17 However, a stent deployed within an infected field has the potential for infection, with devastating consequences. A meta-analysis published on the use of stent grafts for the repair of infected aortic aneurysms reported a graft infection rate of 22%, and 61% of those who had this complication had died at 1 year follow-up.^16^

We do not have access to cryopreserved allografts or aortic stent-grafts on an emergency basis. Considering the patient’s hemodynamic parameters and his physiological reserves at the time of surgery, we felt that time spent harvesting the femoral veins would compromise his chances of survival. After performing a thorough debridement of the infected aorta and the left psoas muscle, we performed an in situ repair using a polyester graft. Once the cultures had isolated Candida, we intended to treat the patient with IV antifungals during the acute post-operative period and to supplement it with long term oral antifungal therapy. The majority agree that prolonged treatment with antifungals should be given after repair of an infected aortic aneurysm due to Candida.^5^^,^ 8 ^,^ 9

## CONCLUSIONS

Mycotic aneurysm of the aorta due to *Candida* infection is rare. It should be suspected when AAA occurs in patients with risk factors such as debility, immune deficiency, or a history of prolonged treatment with broad spectrum antibiotics. Major surgery in such patients can be extremely challenging, especially in resource-limited settings such as ours.
